# LIMPRINT: Health-Related Quality of Life in Adult Patients with Chronic Edema

**DOI:** 10.1089/lrb.2018.0084

**Published:** 2019-04-17

**Authors:** Gregoire Mercier, Jenica Pastor, Christine Moffatt, Peter Franks, Isabelle Quéré

**Affiliations:** ^1^ CHU de Montpellier, Montpellier, France.; ^2^ CEPEL, UMR 5112 CNRS Université de Montpellier, Montpellier, France.; ^3^ School of Social Sciences, Nottingham Trent University, Nottingham, United Kingdom.; ^4^ Centre for Research & Implementation of Clinical Practice, London, United Kingdom.; ^5^ EA 2992 Dynamic Cardiovascular Inconsistencies, Université de Montpellier, UFR de Médecine de Montpellier-Nîmes, Nîmes Cedex 2, France.

**Keywords:** chronic edema, quality of life, lymphedema, lymphoedema

## Abstract

***Background:*** Chronic edema is a condition posing a high burden on patients. The primary aim of the study was to assess the health-related quality of life (QoL) of adult patients living with chronic edema.

***Methods and Results:*** As part of an international, multicenter, prospective study, we prospectively assessed the health-related QoL of adult patients living with a chronic edema using a disease-specific tool and a generic one. In total, 1094 patients were included, aged 57 years on average. The average EQ-5D and LYMQOL visual analogue scale (VAS) scores were equal to 63.6 (SD = 20.2) and 6.3 (SD = 2.0), respectively. After adjustment, the EQ-5D VAS was explained by LYMQOL VAS (β = 7.85; *p* < 0.001), age (β = −0.08; *p* = 0.02), obesity (β = −1.89; *p* = 0.001), and male gender (β = 3.32; *p* = 0.002). As for the LYMQOL VAS, it was independently associated with EQ-5D VAS (β = 0.07; *p* < 0.001), LYMQOL function (β = −0.21; *p* < 0.001), and LYMQOL mood (β = −0.49; *p* < 0.001).

***Conclusion:*** This study confirms that patients living with a chronic edema experience a poor disease-specific and generic health-related QoL.

## Introduction

Chronic edema is characterized by persistent swelling of a body part that has been present for >3 months.^1^ Chronic edema types include lymphedema, lymphovenous edema, lipedema, and gravitational edema. It can affect the upper or lower limbs, the trunk, genitals, head, face, neck, or a combination of these.^2^ Chronic edema is a chronic, incurable condition and poses a high burden on patients due to pain and swelling. Its prevalence has been estimated at 4 per 1000 population^3^; in spite of this high burden, the implications of chronic edema are not well understood.^4^

Published evidence suggests that chronic edema is associated with high levels of functional impairment, anxiety, depression, social impairment, and physical symptoms including discomfort and pain.^5–7^ Poor health-related quality of life (QoL) is related to pain, skin quality, limb mobility, and the frequency of cellulitis/erysipelas and acute inflammatory episodes. Assessing the health-related QoL of patients with chronic edema is of utmost importance to manage patients, to serve as a baseline in the assessment of novel interventions and to develop patient-centered treatment guidelines. However, the vast majority of studies focus on breast cancer–related chronic edema or lower limb chronic edema, and little is known about the impact of chronic edema on health-related QoL.

The present study is part of the LIMPRINT project, an international epidemiological study on chronic edema led by the International Lymphoedema Framework. We hereafter analyze the health-related QoL component of the LIMPRINT project.

The primary aim of the study was to assess the health-related QoL of adult patients living with chronic edema.

## Methods

### Study design and setting

The LIMPRINT study is an international, multicenter, prospective, observational study aimed to determine the impact of chronic edema in adults identified in health services. Patients have been included between 2014 and 2017 in 18 inpatient and outpatient health care centers in six countries (i.e., Canada, Denmark, France, Ireland, Japan, and Turkey). In each center, all consecutive patients aged ≥18 years, having chronic edema for >3 months, and able to understand the study and to give informed consent were included. Patients who were unwilling or unable to participate or were receiving end-of-life care were not included. Chronic edema were identified using a case ascertainment method with one standard questionnaire (CoreTool). The methodology for the overall study is published separately.

### Data collection

Demographic and clinical data collection was conducted using the CoreTool questionnaire. It was completed by a health care professional attending the patient and covers the following 13 domains: type of facility in which data are collected, demographics, level of obesity, mobility, relevant comorbidities, classification of primary or secondary lymphedema, edema history, cellulitis history, categories of treatment, site of swelling, wound area, access to treatment, and subjective control of swelling.

Two health-related QoL questionnaires were completed by the patients themselves: LYMQOL and EQ-5D-3L. LYMQOL is a validated condition-specific QoL assessment instrument.^8^ Separate tools were developed for upper and lower limb lymphedema. The tools are patient-completed questionnaires with 32 items covering four domains: symptoms, body image/appearance, function, and mood. Each item is scored between 1 (not at all) and 4 (a lot). A total score is calculated for each domain by adding the scores together and dividing by the total number of items answered.

In addition, LYMQOL has an overall QoL visual analogue scale (LYMQOL VAS) rating scored 0–10. EQ-5D-3L is a generic QoL instrument applicable to a wide range of health conditions and provides a simple descriptive profile and single-index value for health status.^9^ The EQ-5D-3L comprises the following five dimensions: mobility, self-care, usual activities, pain/discomfort, and anxiety/depression. Each dimension has three levels: no problems, some problems, and extreme problems. For each dimension, the patient's choice results into a one-digit number. The digits for the five dimensions can be combined into a five-digit number that describes the patient's health state.

In addition, EQ-5D VAS records the patient's self-rated health on a visual scale between “100 = best imaginable health state” and “0 = worst imaginable health state.” As recommended for international multicenter studies, utility values were derived using a single-value set, that is, the French one.^10^ All data were entered into a remote data entry application providing a secure online data management system.

### Ethical aspects

The LIMPRINT study was conducted in accordance with current ethical approval and research governance regulatory frameworks applicable to the countries concerned. All patients signed a written informed consent before participation.

### Statistical methods

Quantitative variables were described in the study population with means and standard deviations (SD) or median and first and third quartiles (Q25–Q75) depending on the distribution tested with the Shapiro–Wilk statistic. Qualitative variables were described with frequencies and percentages. Continuous variables were compared with the Student's *t*-test when distribution was normal, or the Mann-Whitney rank-sum test otherwise. For categorical variables, percentages were compared with chi-square analysis or Fisher's exact test. Pearson's correlation coefficients were used to summarize the relationships between continuous variables.

Then, two-multiple linear regression models were implemented to identify the variables independently associated with the EQ-5D and LYMQOL VAS. Only variables significantly associated with the cost difference in bivariate analyses were included in the multivariate models. All statistical tests were performed using a bilateral 5% type-one risk. Statistical analyses were performed with the statistical software SAS version 9.2 (SAS Institute, Cary, NC).

## Results

A total of 1094 patients completed the QoL tools and were included in the analysis. Patients were aged 57 years on average (SD = 14.5), 85.2% were female, 36.1% were obese or morbidly obese, and 85.5% had a secondary edema (Table 1). The average EQ-5D and LYMQOL VAS scores were equal to 63.6 (SD = 20.2) and 6.3 (SD = 2.0), respectively. Both were positively and significantly correlated (ρ = 0.8; *p* < 0.001). Regarding patients with an arm edema, the highest impact was on the symptoms and appearance domains, while it was on function and appearance for leg edema. Overall, the impact on all domains was higher in the case of a leg edema compared with an arm one. LYMQOL domain scores are scaled in a negative direction: the higher the score, the higher the impact and the poorer the QoL. The number of patients by country varied between 24 (Ireland) and 653 (Turkey), and 62.4% of them were included in a specialist lymphedema service (Table 2).

**Table 1.** Patients' Characteristics

	*Mean (±SD) or* N *(%)*
Women	932 (85.2)
Age (years)	57 (±14.5)
Obesity	
Morbidly obese	67 (6.1)
Obese	328 (30.0)
Normal weight	670 (61.2)
Underweight	27 (2.5)
Classification of lymphedema	
Primary	159 (14.5)
Secondary	935 (85.5)
Secondary swelling due to	
Cancer	675 (72.2)
No cancer	260 (27.8)
Lymphedema location	
Upper limb only	565 (51.8)
Lower limb only	525 (48.2)
Lymphedema duration	
<1 year	332 (30.3)
1 year to <5 years	349 (31.9)
5–10 years	170 (15.5)
>10 years	243 (22.2)
Infection last year due to swelling	189 (17.4)
Hospitalization because of infection	119 (10.9)
Swelling and wound	82 (7.5)
LYMQOL	
Arm function	1.73 (±0.62)
Leg function	2.21 (±0.85)
Arm appearance	1.96 (±0.77)
Leg appearance	2.27 (±0.93)
Arm symptoms	2.04 (±0.67)
Leg symptoms	2.13 (±0.81)
Arm mood	1.87 (±0.72)
Leg mood	1.89 (±0.79)
LYMQOL VAS	6.3 (±2)
EQ-5D	
Mobility	1.53 (±0.55)
Self-care	1.42 (±0.57)
Usual activities	1.71 (±0.59)
Pain/discomfort	1.79 (±0.59)
Anxiety/depression	1.61 (±0.63)
EQ-5D VAS	63.6 (±20.2)

VAS, visual analogue scale.

**Table 2.** Distribution by Country and Facility Type

	n	*%*
Country		
Turkey	653	59.69
France	198	18.10
Denmark	115	10.51
Japan	60	5.48
Canada	44	4.02
Ireland	24	2.19
Facility type		
Specialist lymphedema service	683	62.43
Acute hospital outpatient	307	28.06
Other	77	7.04
Acute hospital inpatient	18	1.65
Nursing home	9	0.82

The majority of patients had one of the following specific chronic edema treatments: compression garment, skin care advice, and manual lymphatic drainage (Table 3). More than one third of patients reported at least one barrier in access to specific lymphedema treatment. Overall, 41.1% of patients declared a positive subjective control of swelling.

**Table 3.** Treatment and Disease Control

	n	*%*
Positive subjective control of swelling	450	41.13
Access to lymphedema treatment	316	28.88
Treatment not available for free	216	19.74
Treatment not available within a reasonable traveling distance		
Distance prevents access to treatment	356	35.54
Main treatments		
Compression garment	631	58.00
Skin care advice	578	53.13
Manual lymphatic drainage	547	50.28
Multilayer bandage	478	43.93
Cellulitis advice	347	31.89
Pneumatic compression pumps	184	16.91
Antibiotic	146	13.42
Other complex decongestive therapy	108	9.93
Physiotherapy	86	7.90
Wound dressing	76	6.99
Psychological support	68	6.25
Debulking/lymphatic surgery	11	1.01
No treatment	244	22.43

The EQ-5D-3L VAS was positively and significantly correlated with the LYMQOL VAS (ρ = 0.79; *p* < 0.001). It was negatively and significantly correlated with all the LYMQOL domains with correlation coefficients ranging from −0.46 (function domain) to −0.61 (appearance domain) (Table 4).

**Table 4.** Correlation Between the EQ-5D VAS and the LYMQOL Scores

	*Pearson correlation coefficient (*p*-value)*
LYMQOL VAS	0.79 (<0.0001)
Function	−0.46 (<0.0001)
Appearance	−0.31 (<0.0001)
Symptoms	−0.36 (<0.0001)
Mood	−0.43 (<0.0001)

VAS, visual analogue scale.

In the first multivariate analysis, the EQ-5D VAS was independently associated with LYMQOL VAS (β = 7.85; *p* < 0.001), age (β = −0.08; *p* = 0.02), obesity (β = −1.89; *p* = 0.001), and male gender (β = 3.32; *p* = 0.002) (Table 5). Country, infection, and cellulitis status were not significantly associated with EQ-5D VAS. Hence EQ-5D VAS was significantly higher when LYMQOL VAS was higher, when the patient was younger, nonobese, and a male. On average, a one-point increase in LYMQOL VAS was associated with a 7.85 points increase in EQ-5D VAS; a 1-year increase in age was associated with a 0.08 point decrease in EQ-5D VAS; obese status was associated with a 1.89 point decrease in EQ-5D VAS; and male gender was associated with a 3.32 points increase in EQ-5D VAS. The adjusted model *R*-square was equal to 0.63.

**Table 5.** Variables Associated with the EQ-5D and LYMQOL VAS in Multivariate Analysis

	β	*SE*	p*-value*
Model 1: EQ-5D VAS			
LYMQOL VAS	7.85	0.19	<0.0001
Age (years)	−0.08	0.03	0.002
Obesity	−1.89	0.59	0.001
Male patient	3.32	1.07	0.002
Model 2: LYMQOL VAS			
EQ-5D VAS	0.067	0.002	<0.001
LYMQOL function	−0.21	0.05	<0.001
LYMQOL mood	−0.49	0.06	<0.001

β, regression coefficient; SE, standard error; VAS, visual analogue scale.

In the second multivariate analysis, the LYMQOL VAS was independently associated with EQ-5D VAS (β = 0.07; *p* < 0.001), LYMQOL function (β = −0.21; *p* < 0.001), and LYMQOL mood (β = −0.49; *p* < 0.001) (Table 5). None of the other demographic and clinical variables were significantly associated with LYMQOL VAS. Hence LYMQOL VAS was significantly higher when EQ-5D VAS was higher, and when LYMQOL function and mood were lower. On average, a one-point increase in EQ-5D VAS was associated with a 0.07 point increase in LYMQOL VAS; a one point increase in LYMQOL function was associated with a 0.21 point decrease in LYMQOL VAS; and a one point increase in LYMQOL mood was associated with a 0.49 point decrease in LYMQOL VAS. The adjusted model *R*-square was equal to 0.66.

## Discussion

In this international, prospective, multicenter study on 1094 patients with chronic edema, the average health-related QoL measured with the LYMQOL VAS was equal to 6.3 (SD = 2.0) and the average EQ-5D VAS score was equal to 63.6 (SD = 20.2).

Results from this study suggest that chronic edema has a considerable impact on a patient's health-related QoL. This is in line with several previously published studies in various countries.^1,5,6,11–13^ In a sample of patients living with a lower-limb chronic edema in Ireland, Green and Meskell reported a higher score for each of the four LYMQOL domains (i.e., a poorer QoL) and a poorer overall QoL measured with the LYMQOL VAS score (5.7 compared with 6.3 in our study).^6^ Since there was no major difference in terms of demographic and clinical characteristics, this could be due to the fact that the Irish study has included only patients with lower-limb chronic edema.

Regarding the EQ-5D VAS, the values reported in our study (i.e., 63.6) denote a poor self-reported health. Indeed, for the same set of countries, EQ-5D VAS population norms estimated from national or regional surveys ranged between 76.8 (France) and 83.7 (Denmark) in the general population, and between 74 (Italy) and 81.6 (Denmark) in the 55–64 age groups.^14^ The 63.6 value lies below the 25th percentile for all the available countries (i.e., Denmark, France, the UK, Canada, and Japan) with the exception of Italy. This suggests that patients living with chronic edema are in the bottom 25% of the general population as regards EQ-5D VAS.

Similar results are reported in a case–control study of 107 cases (82% women) matched with 102 age/sex controls in London. EQ-5D health index scores were significantly reduced in the cases (66) by 13 points compared with controls (79; *p* < 0.001). Results from the VAS showed a similar difference (64/76; *p* < 0.001). The mobility questions confirmed the significant impact on mobility with 68 (64%) of cases stating they had problems with walking or were confined to a wheelchair or bed compared with only 36 (36%) of the controls (*p* < 0.001).^15^

EQ-5D results were also similar in a prospective cohort design study involving the intervention of a new service following a 6-month baseline period in the UK. QoL showed greatest improvements between baseline and 6 months post-implementation, the largest differences being in role physical (*d* = 32.7; *p* = 0.0001) and role emotion (*d* = 24.0; *p* < 0.0001). EuroQol increased following the implementation of the new service by a mean score of 0.05 (*p* = 0.007).^16^

In the multivariate linear models undertaken in this study, LYMQOL VAS was significantly higher when EQ-5D VAS was higher, and when two LYMQOL domains (function and mood) were lower. This is consistent with the strong correlation observed between the LYMQOL and EQ-5D VAS scores. Regarding the LYMQOL domains, this result suggests that the function and mood domains are the ones that contribute the most to the overall LYMQOL VAS. Unfortunately, previous quantitative studies did not report such correlation (Greene 2017).

It is interesting to note that, after adjustment, LYMQOL VAS is not explained by any demographic or clinical variable, as in the Irish study (Greene 2017). This suggests that LYMQOL VAS is a very effective way to capture the disease-specific overall QoL. As for EQ-5D VAS, it was significantly higher when LYMQOL VAS was higher, when the patient was younger, nonobese, and a male. The association with age and gender was expected because, in all countries having general population reference norms, the EQ-5D VAS is lower for older people and among women.^14^ Obesity is a condition that is known to be associated with reduced health-related QoL.^17,18^

Patients with a chronic leg edema tended to declare a higher impact of the disease on health-related QoL than patients with a chronic arm edema. This is consistent with the Irish study that suggests a poorer overall and domain-specific QoL in patients with a chronic lower-limb edema.^6,15,16^

This study suffers from some limitations that should be acknowledged. First, the representativeness of the sample might be questioned by the real-world observational design of the study. But the sample is comparable in age and gender with previous work,^6^ and investigators were asked to prospectively include all consecutive patients meeting the inclusion criteria.

Moreover, we included patients with arm, leg, and other localizations of chronic edema in different inpatient and outpatient health care settings. Hence the risk of selection bias was controlled as much as possible. Second, in addition to the disease-specific LYMQOL tool, we used the generic EQ-5D scale. One could argue that it is expected not to accurately reflect the disease-specific health-related QoL; however, we did so purposely to highlight the complementarity between both tools. Nevertheless, this multicenter, real-life, international study has the largest sample of patients with chronic edema among the studies reported so far. In addition, the LIMPRINT study relies on a standardized, clinical definition of chronic edema that reduces the risk of including a patient without a chronic edema.

To conclude, the LIMPRINT study confirms that patients living with a chronic edema experience a poor health-related QoL and that EQ-5D and LYMQOL seem to be complementary tools to assess this population.
